# Supplementary material for: Impaired Nitric Oxide Synthetase Activity in Primary Ciliary Dyskinesia—Data-Driven Hypothesis
Source: J Clin Med. 2023 Sep 16;12(18):6010. doi: 10.3390/jcm12186010 (PMC10531778; doi:10.3390/jcm12186010)
Supplement: Supplementary file 1 [file jcm-12-06010-s001.zip › jcm-2555095-supplementary.pdf]

Supplementary Data

Table S1. Diagnostic parameters of patients with primary ciliary dyskinesia (PCD).

**Diagnostic parameters**	**n**		
Situs	24	Situs inversus	12
		Situs solitus	12
HVMA	21	Immotile	16
		Dyskinetic	5
TEM	15	IDA + ODA defect	8
		ODA defect	1
		IDA defect	1
		Microtubule disorganization	2
		IDA defect + microtubule disorganization	1
		normal	2
IF	11	DNAH5	8
		DNAH5 + DNALI2	1
		DNAH5 + DNALI1	1
		RSPH9	1
Genetics	16	DNAH5	6
		ARMC4	2
		DNAAF3	1
		DNAI2	1
		DYX1C1	1
		SPAG1	1
		DNAI1	1
		CCDC39	1
		CCDC40	1
		RSPH4A	1

Abbreviations. n = number of subjects, HVMA = high-frequency video-microscopy analysis, TEM = transmission electron microscopy, IF = immunofluorescence

Table S2. Data of patients with cystic fibrosis on treatment with CFTR modulators.

**Numbers of subjects**	**25**
CFTR modulator	9 (36%)
- Ivacaftor	2
- Lumacaftor/Ivacaftor	6
- Tezacaftor/Ivacaftor	1
- Tezacaftor/Ivacaftor/Elexacaftor	0

Table S3. Pulmonary bacterial colonization in patients with primary ciliary dyskinesia (PCD) and cystic fibrosis (CF).

	**PCD**	**CF**
Number of subjects	23	25
Pathological bacterial colonization	10 (43.48%)	20 (80%)
- Aspergillus fumigatus	0	1
- Burkholderia cenocepacia	0	1
- Candida albicans	1	0
- Candida tropicalis	0	1
- Enterobacter cloacae	0	1
- Escherichia coli	0	2
- Haemophilus influenzae	5	0
- Haemophilus parainfluenzae	1	0
- Moraxella catarrhalis	1	0
- Methicillin-resistant Staphylococcus aureus (MRSA)	0	1
- Mycobacterium abscessus	0	1
- Neisseria flavescens	2	0
- Neisseria meningitidis	1	0
- Pseudomonas aeruginosa	1	7
- Proteus mirabilis	0	1
- Rothia mucilaginosa	1	0
- Staphylococcus aureus	5	12
- Serratia marcescens	1	0
- Streptococcus pneumoniae	2	0

Table S4. Contents in plasma (µmol/L) and urine (µM or µM/mM creatinine) of metabolites of the L-Arg/NO pathway in patients with PCD or CF and in healthy controls (HC). Data are presented as median [25-75th interquartile range].

**Plasma**	**PCD**	**CF**	**HC**	***p* value**
Number of subjects	22	24	14	
L-Arg	77.14<br>[67.70 – 89.48]	87.56<br>[72.65 – 116.86]	83.45<br>[71.96 – 86.66]	0.110
hArg	1.25<br>[0.93 – 1.70]	1.40<br>[0.93 – 1.79]	1.43<br>[1.10 – 1.67]	0.606
ADMA	0.51<br>[0.36 – 0.55]	0.53<br>[0.46 – 0.59]	0.41<br>[0.36 – 0.52]	0.886
**Nitrite**	23.90<br>[22.19 – 24.92]	22.22<br>[21.45 – 23.62]	21.02<br>[19.65 – 22.64]	* 0.163<br># **< 0.001**<br>+ **0.038**
**Nitrate**	63.26<br>[56.26 – 67.75]	81.61<br>[73.88 – 100.86]	86.96<br>[70.93 – 110.41]	* **< 0.001**<br># **0.002**<br>+ 0.999
**Orn/Cit**	45.25<br>[38.36 – 50.88]	58.03<br>[44.38 – 64.53]	42.17<br>[38.87 – 48.45]	* 0.087<br># 1.00<br>+ **0.046**
L-Arg/ADMA	166.72<br>[144.10 – 199.50]	176.34<br>[136.98 – 222.85]	172.15<br>[158.26 – 234.17]	0.531

Urine				
Number of subjects	24	25	14	
L-Arg	33.17 [18.88 – 43.22]	35.89 [25.68 – 61.10]	32.27 [10.55 – 48.17]	0.145
hArg	2.82 [1.12 – 6.58]	2.10 [1.61 – 3.70]	1.99 [1.10 – 2.72]	0.734
ADMA/Crea	4.08 [3.21 – 5.26]	4.29 [3.41 – 4.99]	3.22 [2.57 – 3.65]	0.077
Nitrite/Crea	0.20 [0.12 – 0.45]	0.22 [0.15 – 0.34]	0.23 [0.15 – 0.66]	0.320
**Nitrate/Crea**	54.84 [44.57 – 82.64]	93.80 [75.54 – 133.66]	74.58 [57.04 – 103.02]	* **0.006** # 1.000 + 0.185
Orn/Cit	42.63 [25.26 – 65.70]	58.86 [34.26 – 73.9]	49.77 [15.09 – 88.32]	0.170
UnoR	311.70 [175.81 – 403.66]	530.48 [268.08 – 655.81]	204.78 [168.53 – 633.59]	0.070

Statistics: Bold indicates statistical significance; * = Significance PCD vs. CF; # = Significance PCD vs. HC; + = Significance CF vs. HC

Abbreviations. L-Arg, L-Arginine; hArg, Homoarginine, ADMA, asymmetric dimethylarginine; Crea = creatinine; Orn/Cit, Ornithine/Citrulline ratio; UnoR = Urinary nitrate/nitrite ratio

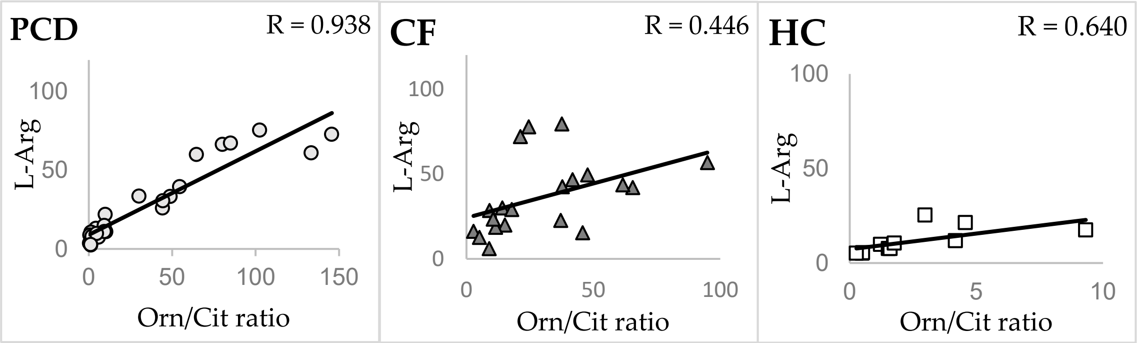

Figure S5. Correlation between L-Arginine (μM/mg sputum) and Ornithine/Citrulline ratio (μM/mg sputum) in sputum of patients with primary ciliary dyskinesia (PCD), cystic fibrosis (CF) and healthy control (HC). R = Pearson correlation coefficient.
